# CATH FunFHMMer web server: protein functional annotations using functional family assignments

**DOI:** 10.1093/nar/gkv488

**Published:** 2015-05-11

**Authors:** Sayoni Das, Ian Sillitoe, David Lee, Jonathan G. Lees, Natalie L. Dawson, John Ward, Christine A. Orengo

**Affiliations:** 1 Institute of Structural and Molecular Biology, UCL, Darwin Building, Gower Street, WC1E 6BT, UK; 2 Department of Biochemical Engineering, UCL, Gower Street, WC1E 6BT, UK

## Abstract

The widening function annotation gap in protein databases and the increasing number and diversity of the proteins being sequenced presents new challenges to protein function prediction methods. Multidomain proteins complicate the protein sequence–structure–function relationship further as new combinations of domains can expand the functional repertoire, creating new proteins and functions. Here, we present the FunFHMMer web server, which provides Gene Ontology (GO) annotations for query protein sequences based on the functional classification of the domain-based CATH-Gene3D resource. Our server also provides valuable information for the prediction of functional sites. The predictive power of FunFHMMer has been validated on a set of 95 proteins where FunFHMMer performs better than BLAST, Pfam and CDD. Recent validation by an independent international competition ranks FunFHMMer as one of the top function prediction methods in predicting GO annotations for both the Biological Process and Molecular Function Ontology. The FunFHMMer web server is available at http://www.cathdb.info/search/by_funfhmmer.

## INTRODUCTION

The rapidly accumulating uncharacterized protein sequences in our databases and their increasing diversity presents new challenges to existing function prediction methods. Functional inference for uncharacterized proteins using simple similarity metrics, for example, by sequence homology search using BLAST (1), can often lead to erroneous functional assignments (2,3). This can arise due to: annotation transfer from paralogs (4), multifunctional proteins (5) or assignments within the twilight zone of protein similarities (6); domain-shuffling in multidomain proteins (7) and misannotations existing in the databases (8). A more sophisticated search for homologs using functional subclassification of the protein domains into functional families can not only help in improving the reliability of function predictions for uncharacterized sequences (9–11), but it can also aid in the identification of highly conserved features of a functional family, which are expected to be functionally important (12). Furthermore, it potentially allows more sensitive detection of new family members by homology recognition, better discrimination between nonmembers and identification of ‘novel’ sequences that do not match any existing families (13).

The CATH-Gene3D resource provides a comprehensive classification of protein sequence and structural domains into homologous superfamilies that have been further subclassified into functional families (FunFams) using the automated family classification protocol, FunFHMMer (14). This subclassification provides a coherent grouping of protein domain relatives into families that can be exploited to predict functions using a domain-centric approach (10,11). Each FunFam is associated with a set of Gene Ontology (GO) (15) annotations associated with the parent proteins of the domain sequences that make up the FunFam. Function annotation using this domain-based approach involves analysis of the domain components of a query protein followed by annotation transfer from the functionally characterized sequences of the family it matches best.

Here, we present a web server that exploits FunFHMMer for functional family assignment and inheritance of functional annotations for query protein sequences. The web server pipeline first identifies the multidomain architectures (MDAs) of query sequences using DomainFinder3 (16) and then assigns GO functional annotations from the best-matching FunFam for each of the domain assignments. The output of the server provides the CATH superfamily and FunFam assignments within the query sequence, highlighting the MDA of the sequence. The GO annotations for each of the matched FunFams are displayed in a table along with their annotation frequency. The GO annotation table can be visualized using the REViGO (17) web server using the link provided.

## MATERIALS AND METHODS

### The CATH-Gene3D resource

The CATH-Gene3D resource (version 4.0) consists of the protein structure classification database, CATH (Class, Architecture, Topology, Homology), which classifies > 235 000 structural domains into 2735 homologous superfamilies based on their evolutionary origin and Gene3D, which populates the CATH superfamilies with an additional > 25 million domain sequences from Uniprot and Ensembl (14,18). The majority of the CATH superfamilies (> 90%) are small in size and the domain relatives share similar structures and functions (10). However, < 5% of the superfamilies are very large, accounting for ∼50% of all domains classified in CATH, and can incorporate a large amount of structural and functional diversity (10,19). In order to understand how function is modulated by sequence and structural changes, and to predict functions in these diverse homologous superfamilies, domain relatives in all superfamilies in CATH have been further subclassified into domain families (FunFams) sharing the same or highly similar functions.

### Functional classification of domain superfamilies

The sequence and structural domains in CATH-Gene3D (version 4.0) superfamilies have been further subclassified into functional families or FunFams using the family identification method, FunFHMMer (14). FunFHMMer subclassifies domain relatives by determining the optimal partitioning of a clustering tree produced with a hierarchical agglomerative clustering algorithm (20). FunFHMMer identifies specificity-determining positions and conserved positions in sequence alignments to calculate a novel index which is used to assess and ensure functional coherence of the identified families (manuscript submitted). CATH currently identifies 110 439 FunFams for 2735 superfamilies. The alignments and profile hidden Markov models (HMMs) for the FunFams are generated using MAFFT (21) and HMMER3 (22), respectively. The FunFams are then associated with a set of high-quality GO annotations from UniProt-GOA (dated 28 May 2013), that are associated with the annotated sequences of the FunFam. GO annotations are considered to be of high-quality if they have: Inferred from Electronic Annotations (IEA) in SwissProt made by either EC2GO or SwissProt Keyword2GO mapping methods, as well as experimentally inferred or curated (non-IEA) annotations in UniProtKB (11). The IEA GO annotations in SwissProt from EC2GO or SwissProt Keyword2GO mapping methods were included since they primarily represent a description of the Enzyme Commission (EC) annotations or manually derived SwissProt Keywords in terms of GO annotations (23).

### The FunFHMMer web server pipeline

**Figure 1.**
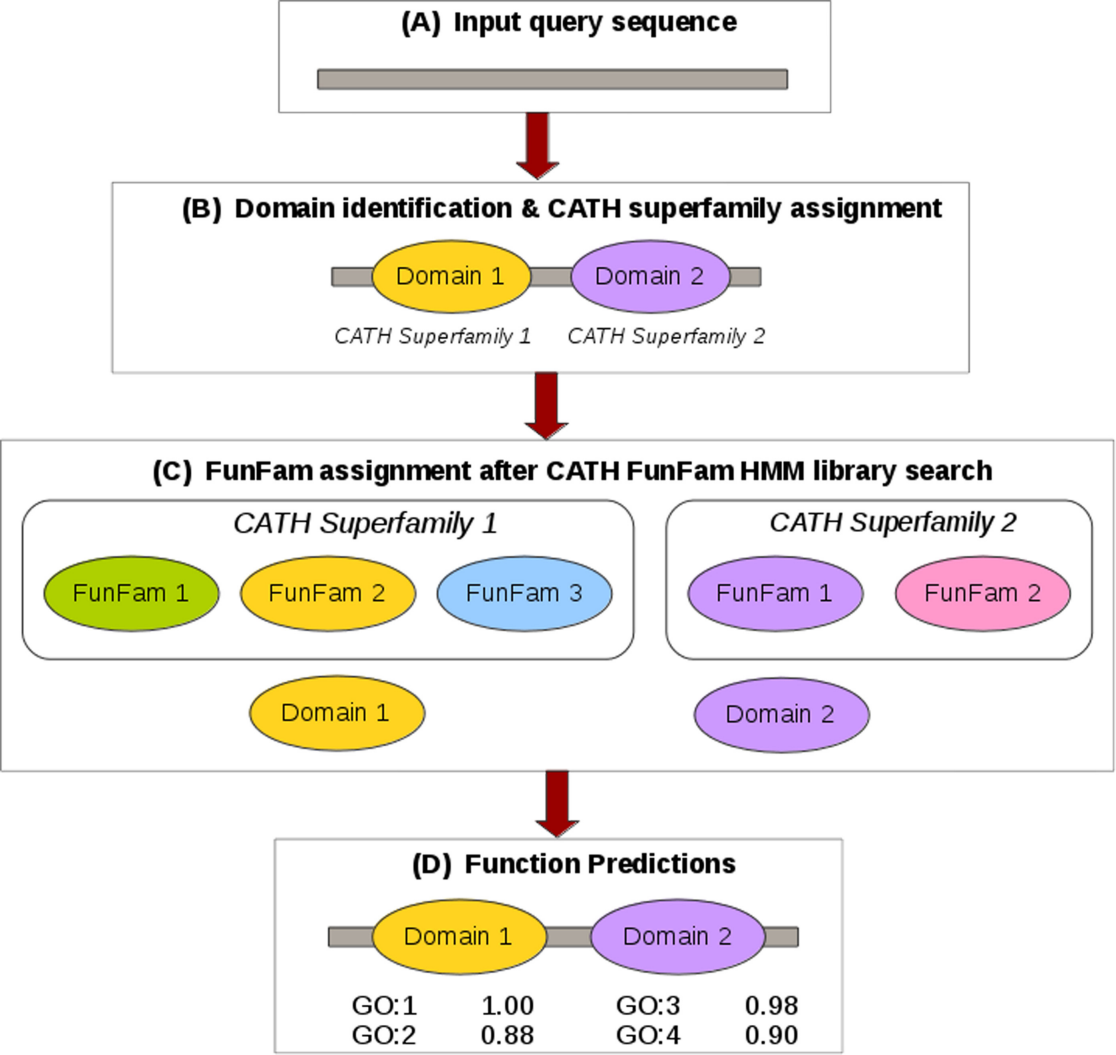
Workflow for the FunFHMMer web server pipeline.

The FunFHMMer web server takes a protein sequence in FASTA format or UniProt/GenBank sequence identifiers as input. The input sequence is scanned against the library of CATH FunFam HMMs using HMMER3 (24). The results are then collapsed into a single set of CATH domain architectures assigned using DomainFinder3 (16) (Figure 1B). Regions on the query sequence are assigned to a FunFam if the E-value of the match to the HMM is significant (i.e. lower than the maximum E-value that is obtained by scanning each sequence within a particular FunFam against that FunFam's HMM (Figure 1C). This maximum E-value is known as the inclusion threshold of a FunFam. The GO annotations for a matching FunFam are then transferred to the query sequence along with confidence scores that are calculated by taking into consideration the frequency of each GO term among the annotated sequences of the particular FunFam. The confidence scores are then propagated up the GO hierarchy or directed acyclic graph (DAG). Finally, a nonredundant set of GO annotations, each GO term retaining its highest confidence score from all the domain regions, making up the GO annotations for the query protein sequence (Figure 1D).

The server is implemented as a Perl-based web application that interacts with a custom distributed queueing system (using beanstalkd as a simple and fast work queue and memcached to provide a distributed storage model for the results). Individual scans should only take seconds and the queueing system enables this performance to scale well.

### Evaluating performance of FunFHMMer

The functional purity of the FunFams generated by our new functional classification method, FunFHMMer, was validated by the Critical Assessment of Function Annotation (CAFA) 2 (2013-2014). FunFHMMer was ranked among one of the top function prediction methods (out of 110 methods) in predicting GO terms in the Biological Process Ontology (BPO) and Molecular Function Ontology (MFO) in the preliminary results of CAFA 2. The preliminary results of CAFA 2 are available here: http://www.slideshare.net/idoerg/cafa-eval. This provided independent validation that the FunFams generated by FunFHMMer are of reasonable purity and are valuable for providing functional annotations for novel, uncharacterized sequences.

The function predictions made by FunFHMMer were also evaluated by comparison against GO term predictions in the MFO obtained from BLAST (version 2.2.29+), the most commonly used tool for function assignment to uncharacterized sequences, Pfam (version 27.0) (25), the most comprehensive and widely used protein domain family resource and CDD (Conserved Domain Database, version 3.10) (26), the meta-protein family resource which integrates curated domain-models and protein-models from various resources including Pfam. This was done by generating a 6-month rollback benchmark test set of proteins. This was a set of proteins that had experimentally-annotated GO annotations in a recent version (28 May 2013) of UniProtKB/Swiss-Prot but which had no functional annotations in a 6 month previous version (25 November 2013) of the resource similar to that used in CAFA (27). Sequence MD5 (a 32 character hexadecimal number) of the benchmark query sequences were used to map sequences between databases (28). Any sequences having higher than 50% sequence identity with any annotated proteins were removed. This was done because these were considered close homologs easy to recognize by BLAST and we wanted to assess the ability of FunFHMMer to predict functions for protein sequences where function annotation transfer from close homologs would be limited. This resulted in a dataset of 557 proteins.

The performance of FunFHMMer was compared to the annotations predicted by: the top annotated BLAST hit for each sequence against the UniProtKB database (dated 28 May 2013), where each MFO annotation is assigned a confidence score equal to 1; Pfam family and CDD family matches where MFO annotations associated with the Pfam/CDD family are assigned confidence scores equal to the annotation frequency of the MFO term among the annotated sequences of a particular family, in a manner similar to FunFHMMer. The confidence scores for GO annotations from BLAST, Pfam and CDD were then propagated up the MFO hierarchy or DAG. For Pfam and CDD predictions, a nonredundant set of GO annotations is then generated for each query protein from the accumulated MFO terms for all significant domain family hits within the query protein in a manner similar to CATH FunFams. The highest confidence score is selected for a particular GO term. (see Section 1.1, Supplementary Material for details). For 281 of these sequences, all the four methods made function predictions. However, only 95 sequences had experimental MFO annotations in the 25 November 2013 version of UniProtKB/Swiss-Prot, as a result, these sequences were used to make up the MFO benchmark dataset (see Supplementary Table in Section 2, Supplementary Material). The performances of the four methods were evaluated using Precision-Recall graphs as in CAFA (2010-2011) (27) (see Section 1.2, Supplementary Material for details).

**Figure 2.**
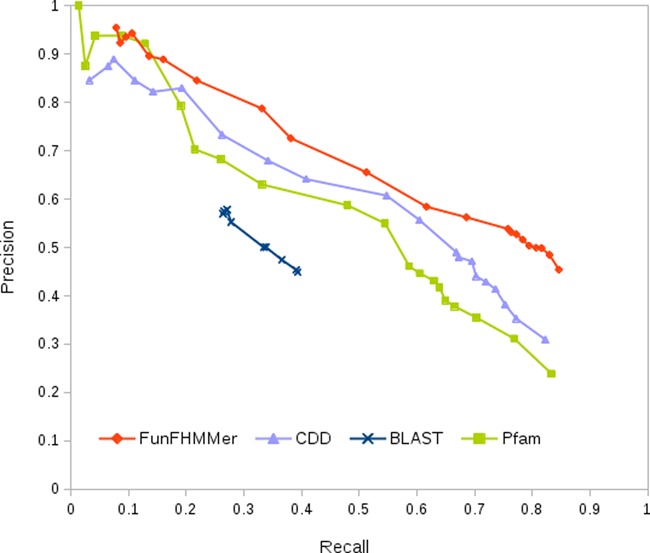
Precision-Recall graph showing the performance of FunFHMMer (shown in red) compared to BLAST (shown in blue), Pfam (shown in green) and CDD (shown in violet) on the benchmark dataset.

The Precision-Recall graph (Figure 2) shows the performance of FunFHMMer compared to BLAST, Pfam and CDD annotation transfer in predicting MFO terms at different confidence score thresholds in the range 0-1. At high confidence scores (> 0.95), which are the most widely used thresholds by biologists, the performance of Pfam, CDD and FunFHMMer are comparable and they all outperform BLAST. CDD performs better than Pfam as it integrates various proteins resources including Pfam. However, FunFHMMer obtains higher precision at all values of recall compared to BLAST, Pfam and CDD. This means that for less confident predictions, FunFHMMer can provide more reliable functional clues for biologists seeking any useful information. This is largely due to the fact that unlike Pfam and CDD, FunFHMMer explicitly classifies sequences using sequence properties related to function.

## THE FUNFHMMER WEB SERVER

### Input

The FunFHMMer web server is available at http://www.cathdb.info/search/by_funfhmmer. The FunFHMMer web server can be queried using a protein sequence in the FASTA format or by entering UniProt/GenBank sequence identifiers as input in the text area on the webpage. The length of the input sequence is not limited and the search for function predictions for query sequences by FunFHMMer is typically very fast (<1 min). However, it may take up to several minutes for very long sequences. A fully documented application programming interface (API) is also provided for interfacing the FunFHMMer search from within any software application.

### Output

The output of the web server provides the MDA of the query sequence along with CATH domain superfamily and FunFam assignments for each domain identified within the query sequence. The EC and GO annotations for each of the predicted FunFams are displayed in tables along with their annotation frequency. The GO annotation table can be visualized using the REViGO (17) web server using the link provided in the results page.

**Figure 3.**
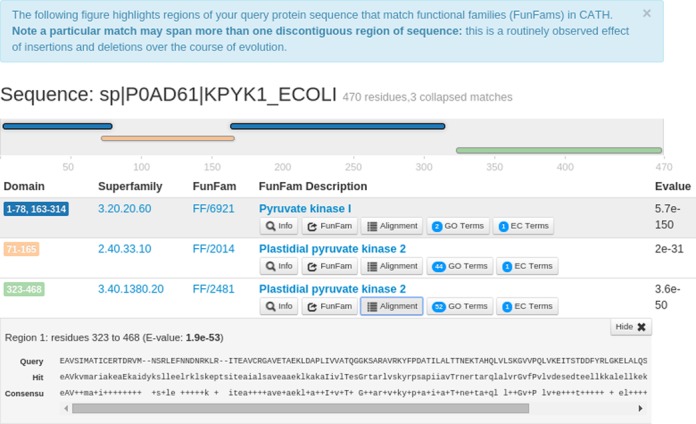
Example FunFHMMer web server results. CATH superfamilies and FunFams (functional families) have been identified within the example UniProt protein sequence P0AD61 submitted to the FunFHMMer web server. Functional information can be retrieved through ‘GO Terms’ and ‘EC Terms’ buttons.

For example, for the UniProt sequence P0AD61, FunFHMMer assigns three structural domains along with their significant E-values (E-values < FunFam inclusion threshold) (Figure 3). The first domain is discontinuous (shown in blue) and matches FunFam 6921 in CATH superfamily 3.20.20.60, the second continuous domain (shown in yellow) matches FunFam 2014 in the CATH superfamily 2.40.33.10 and the third continuous domain matches FunFam 2481 in the CATH superfamily 3.40.1380.20. The description of the FunFams is automatically generated by text-mining the UniProt descriptions of the sequence relatives for each FunFam. These terms may reflect the function of the whole protein rather than the function of the individual domain. For each CATH FunFam match, the ‘Info’ button provides a brief description about the FunFam. To know more about a FunFam, the ‘FunFam’ button directs the user to the CATH FunFam webpage that can provide useful functional and structural information. For example, information on highly conserved positions, highlighted in green, in the FunFam multiple-sequence alignment identified using Scorecons (29) is shown on a representative protein domain structure to highlight residues that are expected to be functionally important.

The Alignment button for each FunFam shows the alignment of the query sequence domain region aligned to the CATH FunFam HMM match using HMMER3. For example, the following figure shows the alignment of the third predicted structural domain in the query sequence (residues 323-468) to FunFam 2481 in the CATH superfamily 3.40.1380.20. The GO annotations and the EC annotations corresponding to each domain are available via the ‘GO Terms’ and ‘EC Terms’ buttons, along with their annotation frequencies. The web server also provides a detailed help page to assist users in understanding the usage of the FunFHMMer web server and its output.

## CONCLUSION

The FunFHMMer web server provides fast, domain-based functional annotations for protein sequences based on functional family assignments in the CATH-Gene3D resource. The web server provides GO annotations for query sequences associated with confidence scores, along with valuable information relating to putative functional sites. The predictive power of the functional families is demonstrated by its recent validation by an international function prediction competition, and its better performance in predicting functions for distant homologs, compared to BLAST, Pfam and CDD on a roll-back benchmark dataset. Moreover, unlike mainly sequence-based resources, FunFHMMer provides direct access to structural information about query sequences, including the 3D location of predicted functional sites identified from multiple sequence alignments of relatives.

## SUPPLEMENTARY DATA

Supplementary Data are available at NAR Online.

SUPPLEMENTARY DATA
