# Supplementary material for: CATH FunFHMMer web server: protein functional annotations using functional family assignments
Source: Nucleic Acids Res. 2015 May 11;43(Web Server issue):W148–53. doi: 10.1093/nar/gkv488 (PMC4489299; doi:10.1093/nar/gkv488)
Supplement: SUPPLEMENTARY DATA [file supp_43_W1_W148__index.html]

CATH FunFHMMer web server: protein functional annotations using functional family assignments — SUPPLEMENTARY DATA 

# CATH FunFHMMer web server: protein functional annotations using functional family assignments

## SUPPLEMENTARY DATA

- SUPPLEMENTARY DATA
