# Supplementary material for: CATH FunFHMMer web server: protein functional annotations using functional family assignments
Source: Nucleic Acids Res. 2015 May 11;43(Web Server issue):W148–53. doi: 10.1093/nar/gkv488 (PMC4489299; doi:10.1093/nar/gkv488)
Supplement: SUPPLEMENTARY DATA [file supp_gkv488_nar-00461-web-b-2015-File004.pdf]

Supplementary Material

CATH FunFHMMer web server: protein functional annotations using functional family assignments

Sayoni Das[1,*], Ian Sillitoe [1,*], David Lee[1], Jonathan G Lees[1], Natalie L. Dawson[1], John Ward[2], Christine A. Orengo[1]

[1]Institute of Structural and Molecular Biology, UCL, Gower Street, WC1E 6BT, UK
[2]Department of Biochemical Engineering, UCL, Gower Street, WC1E 6BT, UK

1 Methods

1.1 Predicting functions of the test set of proteins

The benchmark test set of proteins was generated using a 6 month rollback (May 28, 2013 to November 25, 2013) of the UniProtKB/SwissProt database and proteins were only included if they did not have any annotated homologues with > 50 % sequence identity. The following sections describe the protocols used to predict Gene Ontology (GO) (1) annotations for the Molecular Function Ontology (MFO).

1.1.1 BLAST

BLAST (version 2.2.29+) (2) was used to search for homologous proteins for the test set of proteins against the UniProtKB database (dated May 28, 2013) for homologues of the benchmark proteins. Each benchmark protein is annotated with high quality MFO annotations inherited from the best-matched BLAST hit (using the default BLAST parameters and ensuring that the E-value of the hit is < 0.001) with a confidence score of 1 (see Methods for details). MFO annotations from the UniProt-GOA annotation file (dated May 28, 2013) were used. MFO annotations assigned to the benchmark sequences are then propagated up the MFO hierarchy or directed acyclic graph (DAG). The final confidence scores associated with each MFO annotation after up-propagation are used in the benchmarking (i.e. to derive the CAFA-style (3) plots described below).

1.1.2 Pfam

The benchmark proteins were scanned against the Pfam (version 27.0) (4) family HMM models using HMMER3 (5). The results were collapsed into a single set of Pfam domain architectures using DomainFinder3 (6) and regions on the benchmark proteins are assigned to a Pfam family if the E-value of the match to the HMM is significant (i.e. lower than the inclusion threshold of a Pfam). The benchmark sequences are assigned the high-quality MFO annotations (extracted from the UniProt-GOA annotation file dated May 28, 2013) of annotated sequences in the Pfam

*The authors wish it to be known that, in their opinion, the first two authors should be regarded as Joint First Authors. Correspondence should be addressed to sayoni.das.12@ucl.ac.uk.

family, with a confidence score equal to the annotation frequency of the MFO term amongst all the annotated sequences of that family. This approach is similar to that used for assigning MFO terms and confidence scores to the CATH FunFam matches. The MFO annotations are then propagated up the MFO hierarchy or DAG and the final confidence scores associated with each MFO annotation after up-propagation are used in the benchmarking. A non-redundant set of GO annotations is then generated for each query protein from the accumulated MFO terms for all significant Pfam domain family hits within the query protein. The highest confidence score is selected for a particular GO term.

1.1.3 CDD

The benchmark proteins were scanned against the CDD (version 3.10) (7) family PSSM models using RPS-BLAST with an E-value cutoff of 0.001 and all other parameters as default (8). The results were collapsed into a single set of Pfam domain architectures using DomainFinder3 (6) and regions on the query proteins are assigned to a CDD family if the E-value of the match is significant (i.e. lower than the domain-specific score thresholds used by the NCBI CD-Search (8) tool to determine whether hits to NCBI-curated domain models are specific or non-specific). The query sequences are assigned high-quality MFO annotations (extracted from the UniProt-GOA annotation file dated May 28, 2013) of annotated sequences in the CDD family with a confidence score equal to the annotation frequency of the MFO term amongst all the annotated sequences of that family. This is similar to the approach used for Pfam family and CATH FunFam matches. The MFO annotations are then propagated up the MFO hierarchy or DAG and the final confidence scores associated with each MFO annotation after up-propagation. A non-redundant set of GO annotations is then generated for each query protein from the accumulated MFO terms for all significant CDD domain family hits within the query protein. The highest confidence score is selected for a particular GO term.

1.2 Benchmark evaluation metrics

As in CAFA (3), for each target and some decision threshold $\tau \in [0,1]$, the MFO terms assigned to it with confidence scores greater than or equal to τ, were propagated up the MFO hierarchy or DAG to the root, yielding the set of predicted MFO terms for that target (predicted set). The true MFO terms were extracted from the November 25, 2013 UniProt-GOA file and were also up-propagated the MFO hierarchy for every target (true set). Any terms which overlap between the predicted and the true set were considered as correct at that decision threshold τ. As a result, the precision pr and recall rc for each target were computed as

$$pr_i(\tau) = \frac{\sum_f I(f \in P_i(\tau) \ \wedge \ f \in T_i)}{\sum_f I(f \in P_i(\tau))} \tag{S1}$$

$$rc_i(\tau) = \frac{\sum_f I(f \in P_i(\tau) \ \wedge \ f \in T_i)}{\sum_f I(f \in T_i)} \tag{S2}$$

where $I(f)$ is the standard indicator function, f is a MFO term in the ontology, T_i is the set of true MFO terms (true set) for protein i and $P_i(\tau)$ is the set of predicted MFO terms for protein i with confidence score greater than or equal to τ. f ranges over the Molecular Function Ontology, excluding the root MFO term (GO:0003674). The precision-recall space was then generated by averaging precision and recall across all targets at a given threshold. The average precision and recall at a fixed threshold τ were calculated as

$$pr(\tau) = \frac{1}{m(\tau)} \cdot \sum_{i=1}^{m(\tau)} pr_i(\tau) \quad \text{(S3)}$$

$$rc(\tau) = \frac{1}{n} \cdot \sum_{i=1}^{n} rc_i(\tau) \quad \text{(S4)}$$

where n is the total number of targets, $m(\tau)$ is the number of targets $\leq n$, on which at least one prediction has been made above threshold τ.

Each prediction model was characterized by a precision-recall curve $(pr(\tau), rc(\tau))_\tau$.

2 Supplementary Table

The supplementary table containing the benchmark dataset and the function predictions from BLAST, Pfam, CDD and FunFHMMer can be accessed from `http://release.cathdb.info/v4.0.0/supplementary_files/FunFHMMer_web_server_Supplementary_Table.xls`. Sequence MD5 of the query sequences (a 32 character hexadecimal number) (9) are used to map sequences across databases.

References

[1] Michael Ashburner, Catherine A Ball, Judith A Blake, David Botstein, Heather Butler, J Michael Cherry, Allan P Davis, Kara Dolinski, Selina S Dwight, Janan T Eppig, et al. Gene ontology: tool for the unification of biology. *Nature genetics*, 25(1):25–29, 2000.

[2] Stephen F Altschul, Warren Gish, Webb Miller, Eugene W Myers, and David J Lipman. Basic local alignment search tool. *Journal of molecular biology*, 215(3):403–410, 1990.

[3] Predrag Radivojac, Wyatt T Clark, Tal Ronnen Oron, Alexandra M Schnoes, Tobias Wittkop, Artem Sokolov, Kiley Graim, Christopher Funk, Karin Verspoor, Asa Ben-Hur, et al. A large-scale evaluation of computational protein function prediction. *Nature methods*, 10(3):221–227, 2013.

[4] Robert D Finn, Alex Bateman, Jody Clements, Penelope Coggill, Ruth Y Eberhardt, Sean R Eddy, Andreas Heger, Kirstie Hetherington, Liisa Holm, Jaina Mistry, et al. Pfam: the protein families database. *Nucleic acids research*, 42(D1):D222–D230, 2014.

[5] S Eddy. Hmmer3: a new generation of sequence homology search software. url: http://hmmer. janelia. org. 2010.

[6] Corin Yeats, Oliver C Redfern, and Christine Orengo. A fast and automated solution for accurately resolving protein domain architectures. *Bioinformatics*, 26(6):745–751, 2010.

[7] Aron Marchler-Bauer, Myra K Derbyshire, Noreen R Gonzales, Shennan Lu, Farideh Chitsaz, Lewis Y Geer, Renata C Geer, Jane He, Marc Gwadz, David I Hurwitz, et al. Cdd: Ncbi's conserved domain database. *Nucleic acids research*, gku1221, 2014.

[8] Aron Marchler-Bauer and Stephen H Bryant. Cd-search: protein domain annotations on the fly. *Nucleic acids research*, 32(2):W327–W331, 2004.

[9] Mike Smith, Victor Kunin, Leon Goldovsky, Anton J Enright, Christos A Ouzounis. MagicMatchcross-referencing sequence identifiers across databases *Bioinformatics*, 2116:3429–3430, 2005.
